# Reconstruction of a Thoracic Spine Epithelioid Hemangioendothelioma with Antibiotic Impregnated Poly-methyl Methacrylate: A Case Report

**DOI:** 10.7759/cureus.5713

**Published:** 2019-09-20

**Authors:** Dejan Slavnic, Daniel Carr, Doris Tong, Clifford Houseman

**Affiliations:** 1 Neurosurgery, Ascension Providence Hospital, Michigan State University, College of Human Medicine, Southfield, USA

**Keywords:** spine, thoracic spine, thoracic spine tumor, hemangioendothelioma, epithelioid hemangioendothelioma

## Abstract

A 58-year-old female presented to the hospital with respiratory distress several days after a right hallux amputation. A new lytic lesion within the fourth thoracic (T4) vertebral body and mediastinal lymphadenopathy was noted on chest computed tomography scan. A bone biopsy was performed, revealing bone and collagenous fragments only. Two months later, new imaging revealed approximately 60% lytic destruction of the T4 vertebral body with new right pedicle involvement. Surgical treatment was offered. Intraoperative frozen pathology indicated a hemangioma. An intralesional debulking and stabilization was performed. The right T4 nerve was sacrificed to gain access to the entire vertebral body. Curettage was then used to push the tumor away from the spinal canal into the vertebral body. The spine was reconstructed with 5-10mm beads of Simplex P bone cement (Stryker®, Kalamazoo, MI) which contained 40 grams of poly-methyl methacrylate and 1 gram of tobramycin. Five months after resection, the patient presented with computed tomography and magnetic resonance imaging findings of recurrent disease at T4 and spread to the adjacent T5 vertebral body with lytic changes. At 18 months following her second debulking surgery and radiation treatment, the patient was doing well with no pain or numbness. Long-term imaging compared to the patient’s preoperative imaging displayed improvement in spinal debulking with minimal residual enhancement of tumor despite significant artifact.

## Introduction

Epithelioid hemangioendothelioma was first named by Weiss and Enzinger in 1982 [1]. It has been described as a vascular tumor with malignant potential and the possibility of local recurrence. Hemangioendotheliomas are described as a tumor of intermediate malignancy falling between a benign hemangioma and a malignant angiosarcoma. The epithelioid variant of hemangioendothelioma has the potential for metastasis and is considered the most aggressive of the hemangioendotheliomas. This variant has been noted to occur in liver, lung, skin, and bone as well as other more rare locations [2].

This is a rare tumor with a prevalence of less than one in one million [2]. Optimal treatment of these spine tumors is unknown. Strategies such as intralesional debulking and radiation, en-bloc resection, radiation or radiosurgery alone, and observation have been noted in the literature so far [3]. In light of the rarity of this lesion and the potential for initial underdiagnosis on frozen sectioning, we present a case of a solitary thoracic spine epithelioid hemangioendothelioma with rapid growth over eight months on computed tomography (CT) imaging.

## Case presentation

**Figure 1:**
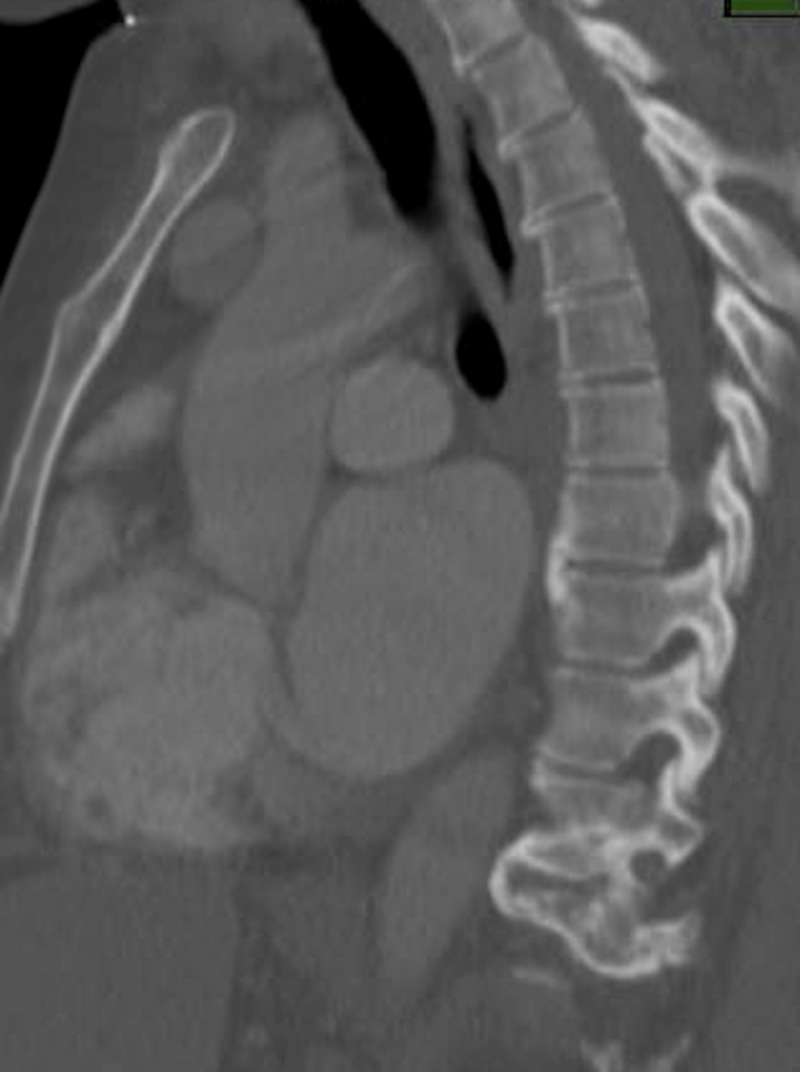
18-month preoperative CT scan depicting no evidence of disease CT (Computed Tomography)

**Figure 2:**
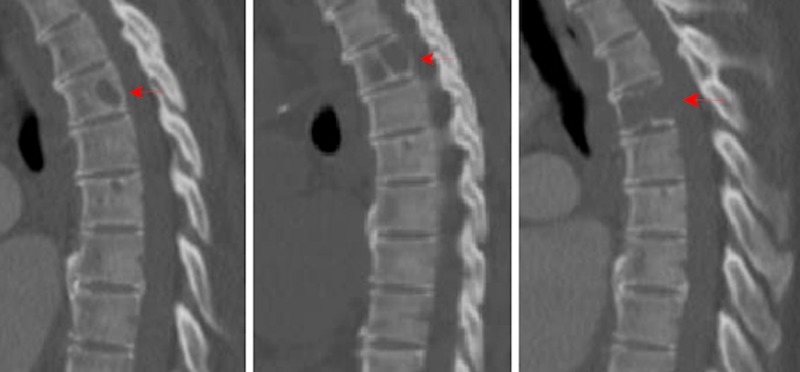
New lesion depicted at T4 with progressive lytic growth and destruction of endplates

A 58-year-old female presented to the hospital with respiratory distress several days after a right hallux amputation. A new lytic lesion was noted within the fourth thoracic (T4) vertebral body and mediastinal lymphadenopathy on chest CT, and previous baseline imaging depicted no evidence of disease (Figures 1, 2). A bone biopsy was performed, revealing bone and collagenous fragments only. The patient returned home and was scheduled for repeat imaging in six months due to the lytic nature of the lesion and a stable neurologic assessment (Figure 2). Two months later, the patient received repeat imaging due to complaints of difficulty ambulating and mid-thoracic pain. New imaging revealed approximately 60% lytic destruction of the T4 vertebral body with new right pedicle involvement (Figure 2).

**Figure 3:**
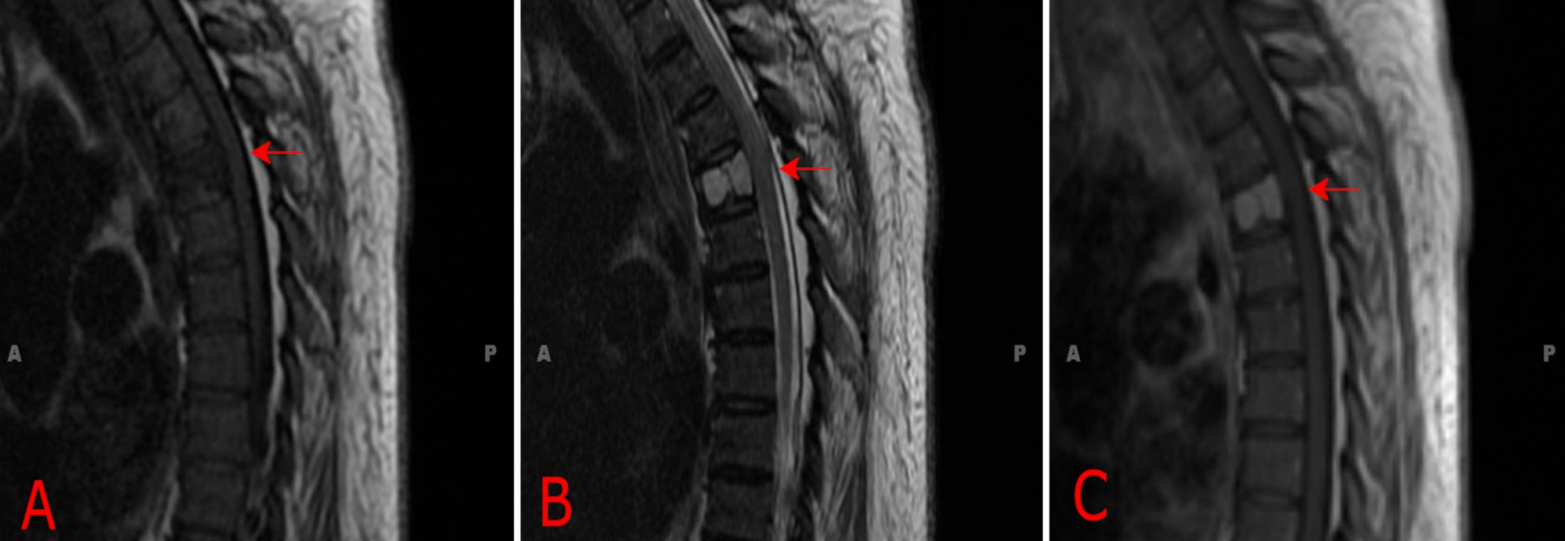
T1-weighted MRI displaying hypointense lesion (A), T2-weighted MRI displaying hyperintense T4 lesion (B), and T1-weighted MRI with contrast displaying enhancement (C) MRI (Magnetic Resonance Imaging)

The T4 lesion appeared hypointense on T1-weighted magnetic resonance imaging (MRI), hyperintense on T2-weighted MRI, and displayed avid homogenous contrast enhancement in T1-weighted MRI with contrast (Figures 3A-3C). There was spinal cord compression ventrally and laterally from right pedicle expansion. Due to the rapidity of growth, the impending likelihood of fracture, and lack of diagnosis, surgical treatment was offered. The lesion had a spinal instability neoplastic score (SINS) of eight out of eighteen [4].

The patient was placed prone on a Jackson table. Uniplanar fluoroscopy was used to confirm the operative level. Standard midline open approach was used to dissect the T3-5 laminae and transverse processes. Pedicle screws (5.5 x 40 mm) were placed at T3 and T5 bilaterally. A complete laminectomy at T4 was performed using a Midas Rex® high-speed drill (Medtronic, Dublin, Ireland). The right facet at T4-5 was removed, and the pedicle at T4 was then biopsied. Purple highly vascular mass was encountered within the right pedicle. Intraoperative frozen pathology was hemangioma.

**Figure 4:**
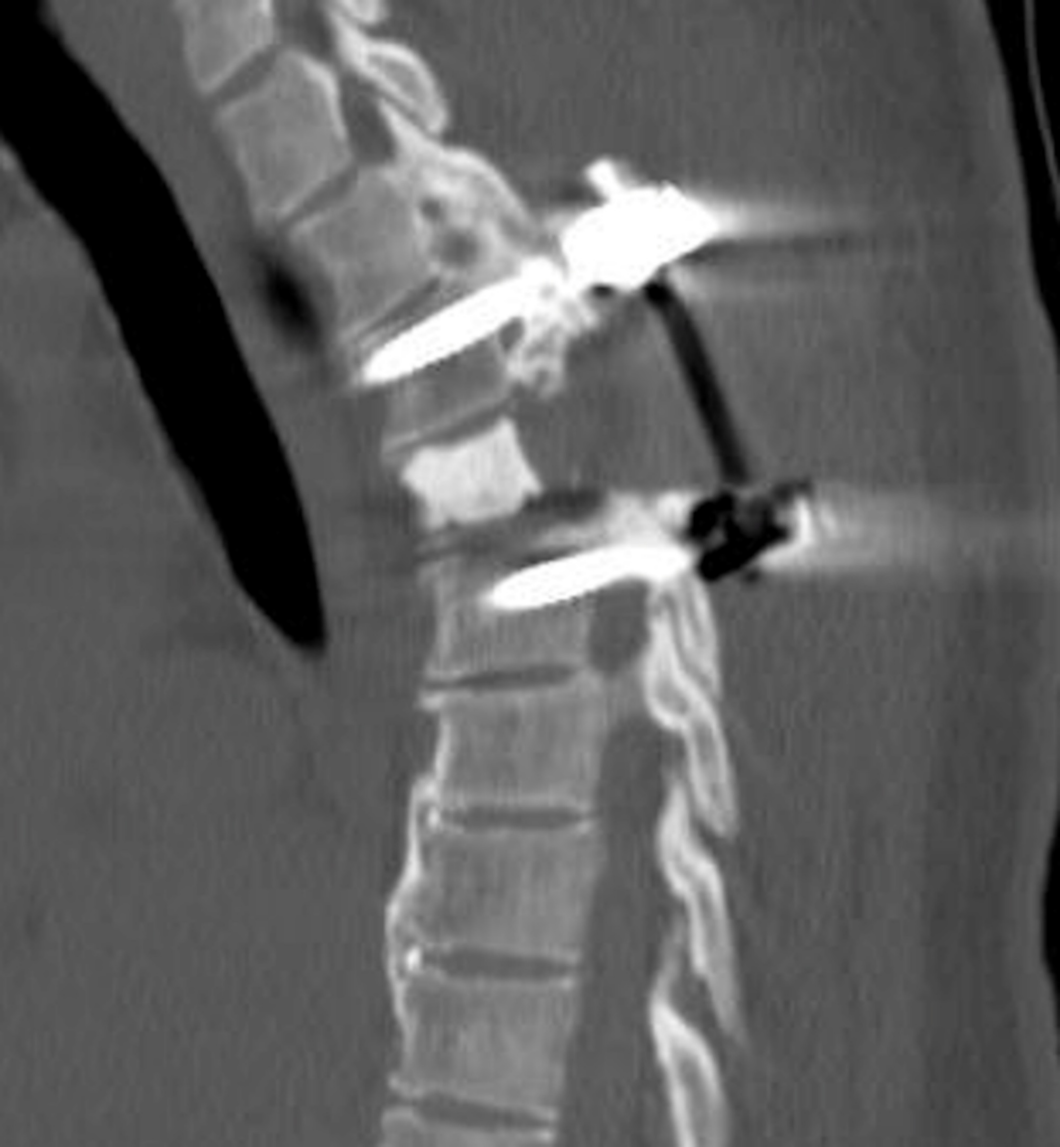
Postoperative CT scan depicting instrumented fusion with anterior PMMA support CT (Computed Tomography), PMMA (Poly-methyl methacralate)

Due to the frozen diagnosis of hemangioma, we performed an intralesional debulking and stabilization rather than an en-bloc resection. The right T4 nerve was sacrificed to gain access to the entire vertebral body. Curettage was then used to push the tumor away from the spinal canal into the vertebral body. Approximately 50% of the vertebral body infiltrated with tumor was removed using curettage. The spine was reconstructed with Simplex P bone cement (Stryker®, Kalamazoo, MI) that contained 40 grams of poly-methyl methacrylate and 1 gram of tobramycin. We introduced 5-10 mm beads of the bone cement into the vertebral column deformity; then before hardening, it was contoured to fit the defect (Figure 4). 

Overall, estimated blood loss for the procedure was 500 mL. There was no immediate or 60-day postoperative complications or neurologic deficits. Final pathology showed epithelioid hemangioendothelioma leading to a plan for radiotherapy as re-resection was deemed high risk due to the patient’s comorbidities and compromised respiratory status. The patient did not comply with the plan to return for radiotherapy.

**Figure 5:**
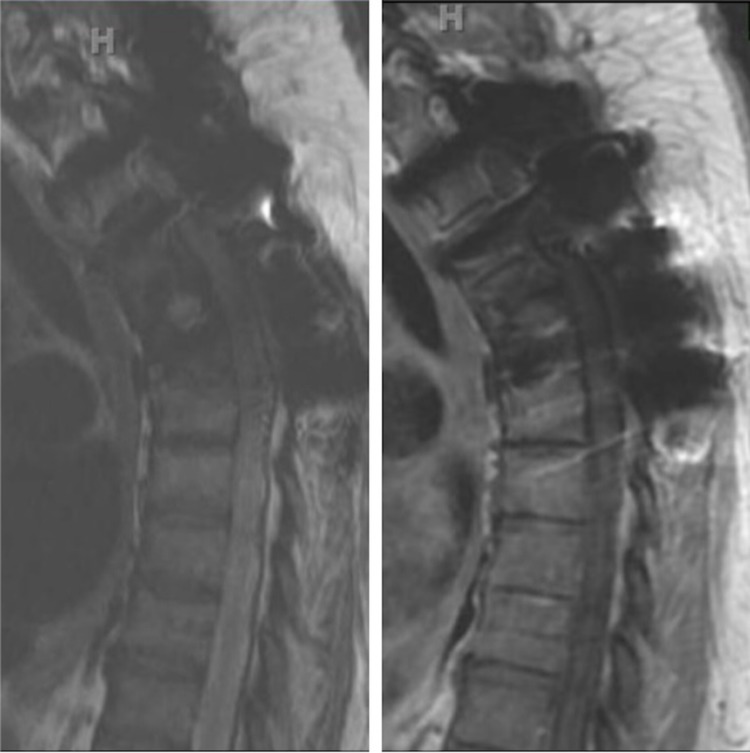
18-month postoperative T2 sagittal MRI (left) and T1 sagittal MRI with contrast (right) MRI (Magnetic Resonance Imaging)

Five months after resection, the patient presented with CT/MRI findings of recurrent disease at T4 and spread to the adjacent T5 vertebral body with lytic changes. At this point, it was decided that the optimal treatment strategy would involve en-bloc resection in an attempt for oncologic cure. However, due to significant comorbidities, we decided that a less extensive surgery involving repeat curettage, debulking, and stabilization of both T4 and T5 expanding lesions could be more safely achieved. The surgery would be followed by irradiation. The patient underwent a T4 and T5 curettage and debulking procedure with an instrumented fusion revision from T2 to T6 with no significant complications post-procedure. Post-operatively, the patient recovered satisfactorily and underwent radiotherapy. At 18 months following her second debulking surgery and radiation treatment, the patient was doing well with no pain or numbness. The patient ambulated with occasional cane-assistance but otherwise had no complaints. Long-term MRI follow-up compared to the patient’s preoperative imaging displayed improvement in spinal mass effect with minimal residual enhancement of tumor despite significant artifact (Figure 5).

## Discussion

While there are many variants of vascular tumors, epithelioid hemangioendothelioma is considered a locally aggressive intermediate malignancy vascular tumor. Considerations for any oncologic resection in the spine include maximal safe resection with reconstruction and stabilization if needed. In specific cases of primary bone tumors, the primary consideration is en-bloc resection with potential for oncologic cure. Our intraoperative frozen diagnosis of hemangioma led to a less aggressive curettage strategy. However, in the case of any rapidly expanding lytic bone lesion on radiography and intraoperative diagnosis of hemangioma, aggressive en-bloc resection may be indicated, since final pathology may sub-characterize a lesion into a more aggressive tumor.

The rarity of this tumor and a lack of evidence-based treatment measures make optimal treatment after surgical resection difficult. Treatment decisions in these rare cases are made based on experience and case-series or -reports. Based on the most recent literature review, no strategy has shown clear superiority. Even with tumor-free margins at the periphery of the resection, there have still been cases of metastasis [3]. There are no pathognomonic radiographic signs of epithelioid hemangioendothelioma tumor on imaging. In the largest case series, six of the ten cases presented were re-operations. Recommendation for wide surgical margins has been proposed but may go overlooked due to incomplete diagnosis from initial frozen pathology [4].

Within the population of those with epithelioid hemangioendothelioma of the spine, there is a variance in response to therapy. A recent case observed an epithelioid hemangioendothelioma to progress extremely quickly while another case observed the same pathology responding well to minimal therapy [5,6]. Kelehan *et al.* reviewed a case of L5 fracture that was diagnosed as an epithelioid hemangioendothelioma [5]. An initial L5 corpectomy of the tumor was abandoned due to excessive bleeding but was subsequently completed after embolization. However, two months after this resection, the tumor quickly progressed to L4 and S1/2 with neurologic decline. Treatment with radiation demonstrated a decrease in epidural compression, but three months after radiation, metastasis to T6 was noted. In contrast, Sebastian *et al. *reported a case of an 18-year-old male with an L2 lesion treated with percutaneous cryoablation, curettage, post-operative Zoledronate, and interferon therapy with no evidence of disease progression at 42 months post-treatment [6]. 

Optimal treatment of spinal epithelioid hemangioendotheliomas eludes physicians at this time. Due to the local aggressiveness of these tumors, aggressive resection and radiation are the preferred choices of treatment. With a rapidly expanding lytic lesion, diagnostic angiography should be considered preoperatively to assess the vascular nature of the tumor. Embolization may or may not be possible based on the proximity to the spinal cord but may offer help in surgical planning. In our institution, we have utilized antibiotic-impregnated poly-methyl methacrylate (AI-PMMA) for the reconstruction of large and irregular spinal defects after large tumor resections. PMMA has a Young’s modulus ten times lower than that of cortical bone and thus acts as an elastic interlayer [7]. The addition of antibiotic was found not to decrease its compressive and tensile strengths and adds an element of safety due to its high tobramycin elution properties and local delivery [8,9]. An additional advantage of applying AI-PMMA instead of titanium cage or another type of strut graft is the exothermic reaction of 70°C to 120°C that is generated during PMMA polymerization which could aid in tumor cell injury and lysis [10,11].

Further studies with long term follow-up are needed to continue to characterize the optimal surgical and post-operative strategies for epithelioid hemangioendothelioma lesions.

## Conclusions

Epithelioid hemangioendothelioma is a rare and malignant vascular tumor with potential for metastasis. The optimal treatment has not yet been elucidated but may include endovascular intervention, radiation, and surgical resection. The ideal surgical technique and approach are mostly surgeon-specific; however, the focus should be on complete resection and potential cure. In our case, the patient was not a candidate for extensive en-block resection due to comorbidities. However, we were able to show good long-term tumor control after debulking, decompression, and spinal reconstruction with AI-PMMA followed by radiation. 
